# Older people care increases the gender gap in academia

**DOI:** 10.1038/s41598-025-13360-1

**Published:** 2025-09-29

**Authors:** María Rosario Vidal-Abarca, Berta Martín-López, Anna Sala-Bubaré, María Anton-Pardo, Nuria Catalan, Anna Freixa, Anna Lupon, Nestor Nicolás-Ruiz, Silvia Poblador, Pablo Rodríguez-Lozano, María del Mar Sánchez-Montoya, María Luisa Suárez

**Affiliations:** 1 Department of Ecology and Hydrology, University of Murcia, Murcia, Spain; 2 Social-Ecological Systems Institute (SESI), University of Lüneburg, Leuphana, Germany; 3 Faculty of Psychology, Education and Sport Sciences, Blanquerna-Ramón Llull University, Barcelona, Spain; 4 Cavanilles Institute of Biodiversity and Evolutionary Biology, University of Valencia, Valencia, Spain; 5 Centre d’Estudis Avançats de Blanes (CEAB-CSIC) Blanes, Girona, Spain; 6 Institut Català de Recerca de l’Aigua (ICRA), Girona, Spain; 7 Centre for Ecological Research and Forestry Applications (CREAF), Barcelona, Spain; 8 Department of Ecology, Autonomous University of Madrid, Madrid, Spain; 9 Department of Ecology, Biodiversity and Evolution, Complutense University of Madrid, Madrid, Spain

**Keywords:** Informal caregiving, Female scholar, Gender equity, Physical and mental health, Professional costs, Quality-of-life costs, Environmental social sciences, Health occupations

## Abstract

Caring for older and for adults with disabilities is nowadays a social challenge for Western societies. However, little research has focused on the role of women as carers of that social group and their personal and professional consequences, particularly in academia. We explore the impact of caring for the older and adults with disabilities on Spanish scholar from a gender perspective. We conducted 36 semi-structured open-ended interviews (24 women, 12 men). Our research finds that, caring for the older and adults with disabilities has costs and implications for scholar on a personal and professional levels, but significantly more for women compared to men. Women often reported more physical and mental health problems than men. In addition, women, reported that their profession was affected by caring for the older people and that they compromised their quality of life mainly in terms of loss of leisure time. Strategies for coping during difficult periods of caregiving differed between genders, whereby women reported reaching out to support networks. At the institutional level, older care is an ‘invisible’ problem, for which interviewees reported not getting any support from their institutions. The study reveals how caring for older people and adults with disabilities adds yet another role and responsibility to women’s dual role as mothers and scholars, leading to “invisible”, often unnamed, impacts on their personal and professional lives: the “triple presence”. Given that the study was conducted in a specific context (Spanish scientists in the fields of environmental science and sustainability), future studies in other geographical areas will be needed to corroborate current findins.

**Supplementary Information:**

The online version contains supplementary material available at 10.1038/s41598-025-13360-1.

## Introduction

There is abundant evidence of women taking the role of ‘caregivers’ at different societal levels [e.g.^1–4^]. Globally, women do 2.6 times more domestic and care work than men^5^. Moreover, women are responsible for 76.2% of all hours of unpaid care work, which represents more than three times the amount done by men^61^. Women continue to do most of the caring duties for family members, while men’s contribution to domestic work remains moderate^6,7^. However, the proportion of men taking on the role of caregiver has recently increased in Western societies^1^. Pacheco et al.^4^ found no gender differences in the time spent on caring duties in Switzerland. Yet, the time spent on caring duties varies according to the family member taken care of. For example, the Barcelona Chamber of Commerce through its Observatory of Women, Enterprise and Economy has indicated that 63% of the informal caregivers of the older adults or a person with disabilities in 2020–21 (August 2020 to October 2021) are women^8^. Different reasons lead women to take the role of informal caregivers of the older people such as altruism, affection, or feeling obligated to take this role due to social norms^9,10^.

This study focuses on a very specific group: “older people” who often suffer from physical or mental problems (“adults with disabilities”) that force them to depend on other persons. Care for older people or adults with disabilities has been recently identified as a societal challenge in Western societies^6,10–12^, regardless of the type of care coverage in different cultural contexts. In the European Union, it is estimated that, by 2030, 21.5 million caregivers will provide weekly care for older people or adults with disabilities for a minimum of 20 h and 10.9 million for a minimum of 35 h per week^13^. Studies on familiar caregiving for older people show that there are several implications of caregiving for older adults, including stress and depression^14^, economic problems^9^, and an overall decrease in their well-being^15–17^. Yet, despite the recent interest in the role of women as caregivers [e g.^1–4^], little research has focused on studying the role of women as caregivers of older people and adults with disabilities and the consequences for them. For example, while the impact of parenthood caregiving duties has been extensively researched in academia [e.g.^3,18–20,32,62^], only a few studies have focused on the caregiving of the older adults^21,22^. Therefore, there is a pressing need to gather information on the personal and professional costs that caring for older.

In this study, we aim to explore the impact of caregiving for the older people and adults with disabilities on academic scholars in Spain. In doing so, we explored the consequences of caregiving on scholars’ professional and personal dimensions, using a gender perspective and a qualitative research approach. This is particularly relevant in the Spanish context because a significant proportion of the academic staff has older parents, since the average age is 49.4 and 54.3 years old for the university and the research centers staff, respectively^23,24^. Moreover, unlike in other countries, care for the older people in Spain has traditionally been carried out within the family^57^.

## Methods

To address the objectives of this study, we opted for a qualitative research approach, which allowed us to delve deeper into participants’ experiences. This type of approach allowed the interviewees to use their own words to freely express what they felt and facilitated the emergence of “unexpected” themes or aspects of the research^25,26^.

### Data collection

We conducted 36 semi-structured interviews to examine participants’ experiences of caregiving and the implications in their professional and personal lives. The invitation to participate was distributed through different scientific platforms, such as conferences and scientific associations, where respondents were connected to or engaged, such as the Iberian Association of Limnology or the Iberian Ecological Society. Once we had a first set of respondents, we applied the snowball sampling technique through which interviewees suggested potential new respondents. We stopped recruiting new interviewees when we reached information saturation, defined as the stage where conducting new interviews yields minimal new information^27^. Most interviews were processed after the interviewee’s intervention, which allowed us to identify the study codes and detect saturation.

During the recruiting process, we made specific efforts to ensure male participation, yet we did not achieve gender parity since women were more willing to participate in this study than men.

The criteria for inclusion of interviewees were: (1) they must have taken care of at least one older people or an adult with disabilities (at the moment or in the past), and (2) they must belonged to academic staff at universities or research centers in Spain at the time they had caretaking responsibilities. This criterion was required because older adults’ care differs according to cultural and institutional contexts [e.g.^14,28^]. Thus, in the context of universities and research centres, caring for older people takes on a different dimension compared to other areas (e.g., administrative and public service staff, medical staff) given the competitive nature of research, which is widely recognized [e.g.^29^], or the intense travelling linked to field work and conferences***.*** Additionally, although we expect similar impacts across countries, we focus in one to avoid biases linked to the idiosyncrasy of the academic structures of each country. So, we narrowed down the sample to the Spanish academic, cultural, and political system.

The participants were 36 Spanish researchers and professors (24 women and 12 men) working in the fields of environmental science and sustainability. All respondents cared for or were caring for older family members, mainly parents. At the time of the interview, 24 out of 36 respondents had cared for older adult’s relatives in the past but not at the time of the interview, while the remaining 12 were caring for older adult´s relatives at the time of the interview. Of the 24 caregivers in the past, 12 had been caregivers for older adults for a long time (between 1 and 12 years).

The average age of the interviewees was 52 years (ranging from 28 to 74 years). Table S1 in the Supplementary Information provides an overview of the socio-demographic characteristics of the interviewees.

The 36 semi-structured open-ended interviews were conducted between July and December 2022, of which 14 were face-to-face and 22 via Zoom. All interviews were audio-recorded after the interviewee’s consent, which was obtained in written form for the face-to-face interviews and via audio recording for the Zoom interviews. All interviews were conducted in Spanish, the native language of all interviewees. Interviews lasted between 10:29 and 44:14 min (average length was 24 min), which in some cases was short and could affect the analytical depth of some topics.

Research ethics clearance was obtained from the Research Ethics Commission of the University of Murcia (Spain) on 26 October 2022 (ID: 4105/2022).

### Interview design

We designed the interview guide according to five sections: (1) personal data and information about their life history, (2) description of the situation of the people under their care, (3) support received from the family and workplace, (4) physical, psychological, professional, and other personal costs of caregiving, and (5) suggestions for improving the conditions in academia during caregiving. Text S1 and S2 in the Supplementary Information present the interview guide in English and Spanish, respectively.

### Data analysis

All interviews were audio-recorded and later transcribed. Before starting the coding process, we defined five dimensions of the research as streamlined in the interview guide: (1) caregiving responsibilities, (2) implications and costs of caregiving, (3) coping strategies, (4) responses given by the academic institutions, and (5) suggestions for facilitating caregiving in academia (Table 1). For analysing the data for each of these dimensions, we conducted a content analysis engaging in an inductive and reflexive approach^30,31^.

**Table 1:** Dimensions and themes identified in the study.

Dimension	Themes
1. Caregiving responsibilities	Reasons to become the main caregiver Who helps the caregiver Getting prepared as a caregiver
2. Implications and costs	Emotional costs Physical and mental health costs Professional costs Economic costs Costs on other dimensions of quality of life
3. Coping strategies	Seeking for help Learning from experience
4. Responses from academic institutions	Institutional support
5. Suggestions	Support and facilitation of caregiving in academia

The coding process was carried out manually following an iterative coding process of reading and rereading the transcribed texts, whereby the first author identified the main codes that emerged for each of the five dimensions mentioned above. The codes that emerged were then discussed among several co-researchers to ensure that there were no overlaps and that the codes covered all of the interviewees’ responses and the five dimensions. Finally, the codes were grouped into the themes presented in Table 1, covering the five analytical dimensions.

During the process, the total number of sources (i.e. the number of respondents) and references (i.e. the number of quotes) for each category were recorded. Frequency (i.e. the number of times a theme was coded in all interview transcripts) was used to indicate the importance participants attributed to the specific theme.

## Results

### Caregiving responsibilities

#### Becoming the main caregiver

More than 45% (11/24) of the interviewed women specifically showed their acceptance of caregiving as a natural fact. For women it is an unavoidable fact of life: “…*to take care of the parents when they are in their last stage because it seems to me that it is life´s law…*” (W19). In contrast, for some men, caring for the older persons to be an imposed obligation: “*It is an additional burden, it is an obligation…*” (M10).

However, some women questioned the socially imposed role of caregiver, including the demands of parents who want to be cared for by daughters: “*They* ⟮parents⟯ *consider that daughters are the ones who really look after them best. When I had not been with them for two weeks, they considered I had abandoned them, even if my brother was looking after them.*” (W13). This quote shows that the care usually fell on the daughters (7 women reported this), even when there are male siblings. This result is illustrated by the following quote: “…*the brother is the one who doesn’t take care of anything. The gender role is totally fulfilled and sometimes it seems unbelievable how this normality is assumed within the family…, even by parents*” (W35).

#### Helping the caregiver

More than 72% (26/36) of all respondents received help from other family members in caring for the older people and adults with disabilities, but the proportion was unequal: while 11 out of 12 male respondents were supported by their family, only 15 out of 24 women obtained support from the family. Moreover, 13 women and 4 men declared that they had to find help outside the family by, for example, hiring professional caregivers or registering the older adults in nursing and residential homes. These practices allowed respondents to actively engage in their work and to relieve some of the caring pressure, as shows the following quote: “*From Monday to Friday, I was helped by the girl who came to stay with her, watching her and taking care of her, while I was working.*” (W29).

Some interviewees emphasized the caregiving challenges when they overlapped with childcare responsibilities—e.g., *“I’ve had to do both things* [looking after parents and children]. *You come home after spending the whole weekend looking after your parents, and you can’t imagine how you find the house with the teenagers*”. (W19).—and when the caregiver is of advanced age—e.g. “*With a young child you have a lot of work, but you are younger, and you have more energy. Whereas with older people, well, it’s a care that consumes more energy than the one that you actually have”* (W19)*.*

#### Getting prepared as a caregiver

Very few participants felt prepared to deal with the care required by the older people and adults with disabilities, especially when it comes to degenerative processes or psychological disorders (8/12 men and all women did not feel prepared). Many of them said that they had to learn as they went along the process by researching and asking acquaintances and professionals. But above all, they stressed that they did not feel emotionally prepared for the task, especially when it entailed long-term caregiving: “*…I was not prepared and everything is a daily learning process… in my mother’s case, as it is a long process of continuous degradation, the strategies you use to do certain activities with her, doesn’t work the following week and you have to change… And indeed, I find myself a little lost…*.” (M14).

### Implications and costs

#### Emotional costs

Most of the interviewees (21 women and 11 men) expressed unpleasant emotions. The reported unpleasant emotions included feeling (1) obliged—e.g., “*It is an additional burden, it is an obligation… with few satisfactions, well few, because in reality most of the time they are not very grateful and they demand a lot*” (M10)–, (2) frustrated—e.g., “*I’m not satisfied because I think I could have done it better and given them more love*” (W36)–, (3) guilty—e.g., “…*it’s true that there is a feeling of guilt… guilt for not being there more, for not dedicating more time to her. The guilt of thinking what if I don’t call her every day and tomorrow she’s worse. The guilt is there permanently.*” (W30)–, and (4) sad—e.g., “*I was sad. The weekend I had to go, I knew it would be hard to see how they have been [deteriorated]. And when I came back, I came back crying.*” (W21)–.

Moreover, some interviewees (21 women and 6 men) reported emotional ambivalence because, although they found caregiving to be meaningful, they would like to recover their lives: “*On the one hand, the satisfaction of helping them, and on the other hand, I felt bad about myself because I want this to end and go back to my life.*” (W33). A small minority (3 women and 1 man) did not report negative feelings and explicitly reported pleasant emotions derived from caregiving.

#### Physical and mental health costs

**Fig. 1:**
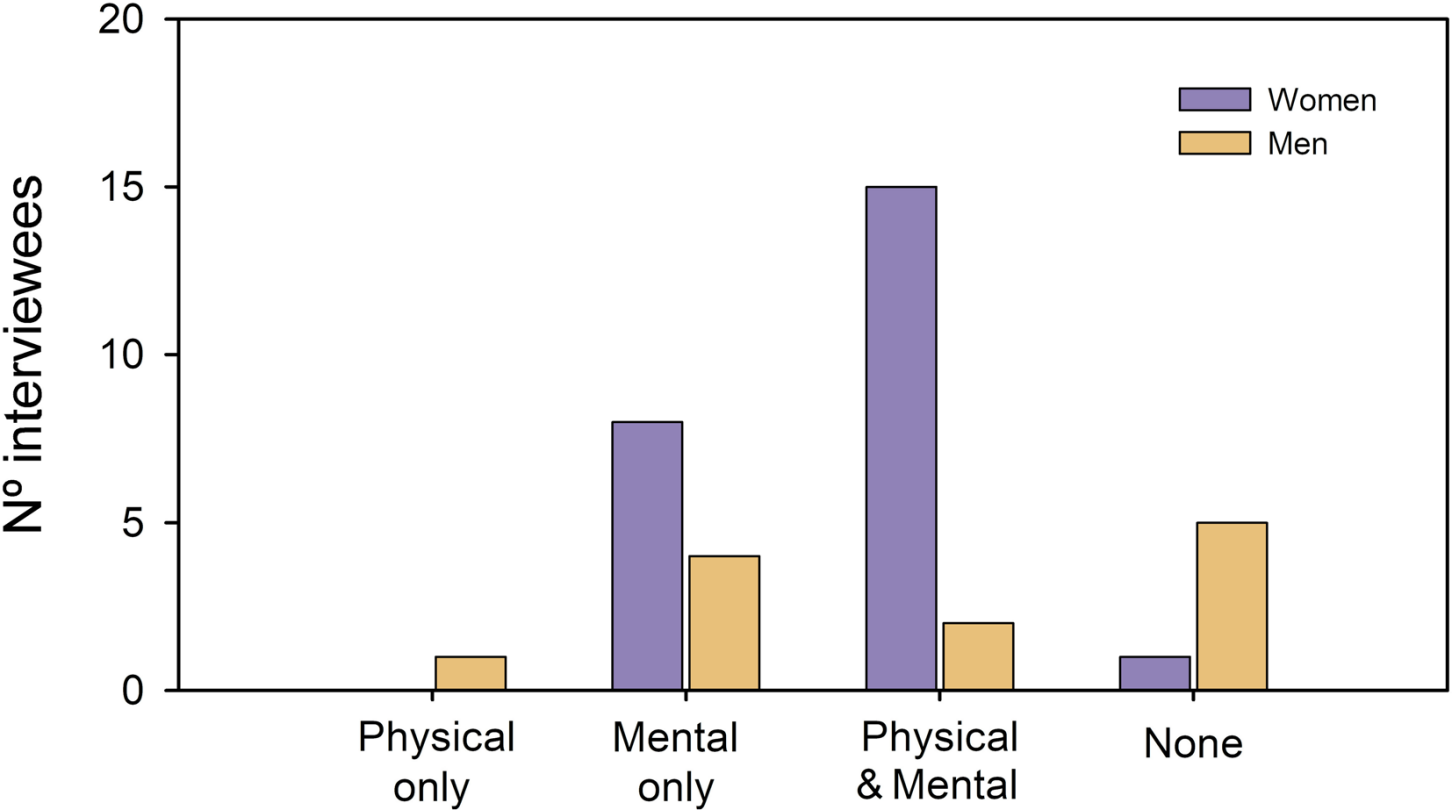
The number of interviewees (women/men) suffering from physical and mental health problems because of caregiving for the older and adults with disabilities.

Half of the respondents (18 interviewees) reported physical health problems, being proportionally more reported by women (63%; 15) than men (16,7%; 2) (Fig. 1). The physical health costs were more diverse for women than men, including back pain and lumbago (3 women), migraine (2), muscle contractions (2) and neck problems (2), among others. Men mainly reported physical consequences related to the stress derived from caregiving: “*A little while ago I had one of these alopecia areata for the first time in my life (as a result) of stress*” (M09).

More than 78% of respondents (28 interviewees) had experienced mental health problems, but the proportion of women (23 out of 24) outnumbered the one of men (5 out of 12) (Fig. 1) and the mental health problems were more diverse for women than men. While women reported stress (15 women), anxiety (7), lack of concentration and psychological exhaustion (4), and depression (3), men only reported about stress.

Interestingly, no women reported having suffered only from physical illnesses (Fig. 1), and 15 women reported having suffered from both physical and mental health problems (Fig. 1). Five men experienced neither physical nor mental health problems (Fig. 1).

#### Professional costs

**Fig. 2:**
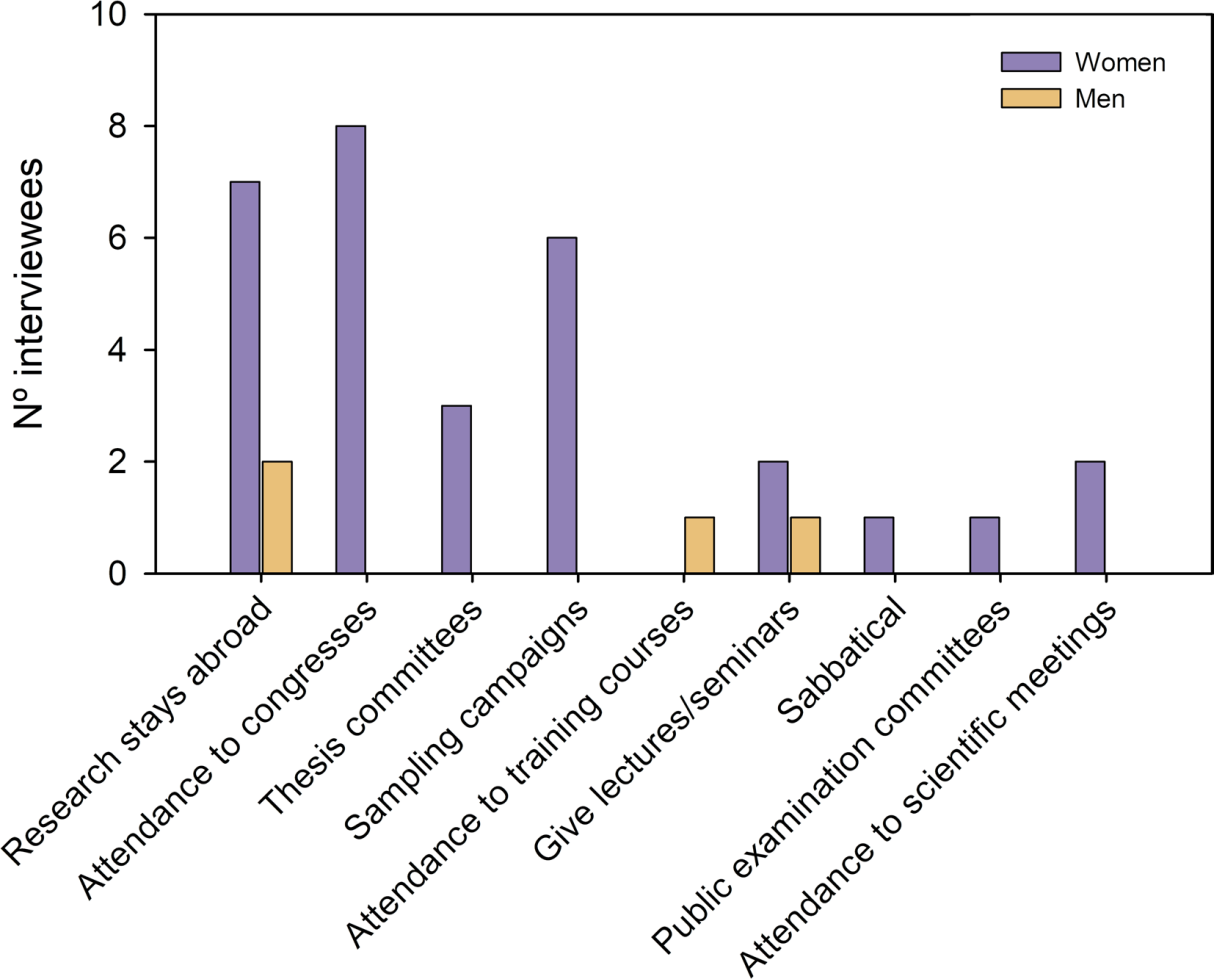
The number of interviewees (women/men) giving up different professional opportunities due to caregiving for the older and adults with disabilities.

Caring for the older people and adults with disabilities involved a professional cost for both men and women, with more women (83%; 20) than men (58%; 7) reporting that their profession was affected (Fig. 2). Often, the professional cost was aggravated with childcare, leading to a reduction in scientific productivity: “*In the first stage of care from 2010 to 2013, which was combined with caring for my daughter, the issue of caring for my father was the straw that broke the camel’s back and I think I lost a lot of scientific productivity there.”* (W35). Yet, when caregiving for the older people did not overlap with childcare, interviewees did not report a decrease in scientific publications, often because the time spent in caregiving was compensated with leisure time or because the writing took place in hospitals and home residences: “*In terms of papers, I don’t think it affected me because I was very clear about what I had to publish. I kept up the pace even when I was in hospital. I wrote several of the publications of the PhD from the hospital.*” (W01).

Professional costs were diverse (Fig. 2), including giving up career opportunities (10 women and 4 men), such as research stays and sabbaticals abroad, conferences, and training courses: “*I no longer consider being a researcher abroad because I don’t want to be too far from my mother.*” (W36). In the case of university professors, teaching was not jeopardized, which was often accomplished at the cost of scientific production and leisure time.

We found differences in the professional costs reported by younger and older generations of scholars. While early career researchers reported that caregiving duties harmed their opportunities to get a permanent position since they had to give up tenure tracks, senior scholars reported that caregiving jeopardized their ability to keep up the pace in science. Moreover, only two interviewees (1 junior and 1 senior) requested to work part-time, while early career researchers (1 woman and 2 men) did not dare to ask because of their fear of losing their grants and contracts: “*I didn’t even think about temporary leave or anything. I was afraid that everything would take longer or that I don’t know to what extent they can tell me that they won’t accept this* [a part-time contract]*. I’m afraid.”* (M15). In addition, some interviewees expressed their concern about going part-time because a reduction in their salary will aggravate the economic costs (see below): “*In theory, a reduction in working hours is contemplated to care for the sick, which is also a salary reduction and if you must pay a professional caregiver, then you need the whole salary. In other words* (…) *it’s not affordable.*” (W16)*.*

#### Economic costs

Twelve interviewees (9 women and 3 men) reported that caring for the older people and adults with disabilities entailed financial costs. The economic costs were mainly associated with obtaining support from external caregivers: “*Half of my salary goes on caring for my mother. She earns very little, and half of my salary goes on professional caregivers and the one who cleans her house.*” (W17).

#### Costs on other dimensions of quality of life

Thirteen interviewees (10 women and 3 men) reported that leisure was the first aspect of their quality of life that was compromised with caregiving for the older people and adults with disabilities, giving up or significantly reducing the time they spent in leisure activities: “*We gave up going on holiday because we have always been a very close family, we did everything together and if in the last few years they were no longer in a position to go on holiday, we didn’t go.*” (W03).

Moreover, seven interviewees (6 women and 1 man) stated that caring for the older people has disrupted their family plans: “*We couldn’t move in together. He lived in a flat on his own. And he went to live with his mother, and I went to live with my mother for two years. In terms of intimate relationships, it has limited us* [as a couple] *a lot.*” (W24).

Loneliness was also reported as a cost to the interviewees’ quality of life. Three interviewees (2 women and 1 man) mentioned that caregiving was something that they could not share with other people, which led to a deep sense of loneliness: “*This is a bit of an illness that is suffered in silence, and besides, I feel uncomfortable asking for help for a problem that is my problem, right? Why do I have to ask for help when many of my colleagues are in the same situation and they solve it on their own?*” (M14).

### Coping strategies

#### Seeking for help

Interviewees used different types of strategies to cope with the challenge of caregiving for the older people and adults with disabilities, including psychological therapy (3 women and 1 man) and antidepressant drugs (4 women): “*…I am taking medication… I went to the psychiatrist because I needed chemistry to go out, I was crying all day… the psychiatrist prescribed me some pills and now I feel better, but I am still not one hundred percent.*” (W17).

Women interviewees also used self-help strategies (6), such as yoga, dance, meditation, pilates and other forms of physical exercise: “*I started to investigate tools to take care of myself, like meditation, biodance, and non-violent communication.*” (W34).

Women interviewees also reported relying on their support network (5), including girlfriends, relatives, and social networks, to overcome the more depressive or stressful phases of caregiving: “*The three of us* [the interviewee and her two aunts] *who care for her* [mother] *do a lot of therapy with each other. I also turn to my support network a lot*” (W18). Some interviewees specifically referred to women`s organizations in science as a source of support: “*I get a lot of help from the Gender Group I am in. In the group there have been a lot of people I have been able to share with and I have found a lot of support there.*” (W36).

Interviewees (7 women and 3 men) expressed gaining support from co-workers, colleagues, members of their research team, and supervisors: “[I gained support] *from my colleagues, yes. By my thesis supervisors, too. Well, by the department in general. They immediately understood me, they empathized with me.*” (M08).

#### Learning from the experience

A few interviewees (4 women and 2 men) expressed that caring for the older people and adults with disabilities has helped them to learn new skills—”*I just got my driving license at the age of 40, I just got it. I just got it to help her* [his mother]” (W05)- and to gain perspective in life—”*It has changed my perspective* [regarding work]*.* (…)* Maybe I have become more of a ‘caring’ person. Before, I think I was more abrupt, more imposing, and now I think I am a bit more serene*” (W06). The reference to a new perspective in life was often mentioned alongside becoming familiar with death: “*Thanks to caring for my older, I am more empathetic, more human and I believe that everyone should care more. Caring for older people prepares you for your end, you become more empathetic. Psychologically, it prepares you for that thing that in our Western society is so dramatized and so taboo, which is death. And I think it* [caring for older people] *has a lot of positive psychological effects.*” (W35).

### Current responses from academic institutions

All interviewees (36 respondents) indicated that they were unaware of the existence of any support in their institutions and that, if there was any, it was ‘invisible’. Interviewees felt that institutions are unaware of the challenges that caring for the older people and adults with disabilities pose on their career and wellbeing: “*Let’s say it is a problem that is not assumed* [by the institution] *and that it becomes our problem.*” (M11). At the same time, interviewees did not consider that they could communicate their situation as caregivers to the academic institution: “*I haven’t even thought about it, I mean, the thing is that you don’t look for support … because you don’t even know that you can get it … I’ve never thought about it, I just haven’t thought about it…*” (W18).

Five interviewees indicated the existence of psychological cabinets in their workplaces; however, they expressed that the psychological support is mostly focused on work-related problems, such as burnout—e.g. “*Here, what the psychological health office works on most is the issue of burnout, people who are burnt out from work.*” (M04) and on helping students—e.g. “*Now I know that the psychology support service of the university does workshops for students but not for us* [academic staff]*.*” (W24).

Eight interviewees (6 women and 2 men) specifically point to the flexibility of academic work since it often does not require a continuous presence in the workplace: “*The university allows quite a lot of flexibility in the way of working, in the timetables and, for example, in the organization of the distribution of the teaching load among the colleagues. But I think that this flexibility makes it* [referring to the care of the older people] *invisible.*” (M32).

### Suggestions by interviewees to mitigate the caregiving costs

The interviewees provided some ideas that could improve the situation of balancing caregiving with an academic job. One woman pointed out the possibility of considering teleworking as an option to better reconcile teaching and research workload with caring for the older people and adults with disabilities. However, at the same time, they also recognized that this could become a trap where scholars do not stop working: “*…in the end, it ends up being a continuous 24-h working day*” (M14).

A second suggestion mentioned by two interviewees (1 woman and 1man) was that academic institutions provide specific psychological help for scholars who are also in caregiving duties: “*At an institutional level there could be some psychological help.*” (M15).

A third suggestion expressed by three interviewees (1 woman and 2 men) was to formally reduce the working hours or to go on leave (i.e., a compassionate or dependency leave) for those scholars who are acting as caregivers without compromising their salary: “*that there is a possibility that at a given moment if someone wants to exercise the role of a caregiver, they have the possibility and institutional support*” (M08).

Finally, three women suggested the creation of ‘day centers’ for the older people adults who are relatives of the academic staff. These centers could also serve as centers where research and teaching related to aging and caregiving take place: “[Referring to a senior center] *With all the professionals that there are in the University who are dedicated to this type of things, it would even serve as a center for them to do research and internships.*” (W33).

## Discussion

In academia, gender biases are well known [e.g.^32–35,63^]. Gender inequity in academia has resulted in lower scientific productivity of women due to their family responsibilities^36^, women’s reduced professional networks^37^, a higher number of unfavorable reports for their work and research proposals^38^, or longer time spent on teaching and service work [e.g.^37,39,40^]. Despite the extensive research on gender inequity in academia, women older adults’ care is rarely highlighted as an important factor that contributes to the gender gap but see^7,21^. However, this issue is not new. For example, the work of Martínez-Angulo et al.^41^ on nurse-patient interactions demonstrates how gender asymmetries and excessive workloads silence the voices of older women and normalise female care work.

### The “triple presence” hardship

Our research demonstrates that more female than male scholars take caregiving responsibilities for the older people and adults with disabilities as a ‘normal’ activity, often driven by the belief of being a moral duty^2,3,42^ and the social expectation of women becoming compassionate and altruistic caregivers^3,14^. Yet, our research shows that acting as caregiver of the older people and adults with disabilities is considered by some female scholars as unfair, a feeling that is aggravated when other family members are not willing to share the task. The social expectation for women to act as caregivers of the older people is also demonstrated by the fact that when female scholars obtain help from the family, this comes from close female relatives (e.g. aunts).

The gender gap in academia amplifies when the female scholars belong to the “sandwich generation” (aged 45–64^43^;) since the caring duties expand from caring for their children to caring for their older (mainly parents), while keeping up the pace at work^44^. According to the Barcelona Chamber of Commerce report^7^, in Catalonia, women of the sandwich generation spend 6 h more per week than men caring for the older people. In our study, the female scholars who belong to the sandwich generation reported several consequences for their careers. The expectation of women to become caregivers for the older people and their children while being scholars can be described as the burden of women’s “triple presence” (after the notion of “double presence” coined by Balbo^45^) as daughter caring for her parents, mother caring for her children and scholar.

Caring for older people and adults with disabilities is not harmless for the physical, mental and psychological health of the caregivers^46^. Women have been reported to suffer more negative impacts than men on their health due to their work as caregivers, reporting 41% significantly more often than men (27%)^8^. Our research aligns with these results. Fifteen out of 24 women reported having physical problems, more than twice as many as the men interviewed (3 men out of 12). However, these figures soar when it comes to mental health since almost 100% (23 women out of 24) of female scholars reported suffering from mental and psychological problems, while only five men out of 12 reported similar problems. It is not surprising that most of the complaints reported by women relate to their mental health given that women are more emotionally involved than men in caring for the older people and adults with disabilities^47^. This emotional involvement is also reflected in our results. 67% of female scholars expressed that, although they accepted caring for their older people, they felt frustrated and guilty for not having enough time to care for them and for not knowing how to understand what they needed. An emotional burden that adds up to the emotional burden of trying to be productive or keeping the pace in academia while caregiving, a burden previously described for female scholars on maternity leave^48^.

### “Superpowers” of female academic caregivers

A differentiating circumstance that makes caregiving particularly relevant to research in academia with a gender lens is the neoliberal academic culture of labor^48,49^. Work in academia demands scholars “all-consuming passion and commitment”^49^^, p. 147^. The manifold tasks required in academia, such as teaching, research, attendance to international meetings and conferences, networking and administrative duties have led to endless schedules^50^ of academic ‘superheroes’^29^. In addition, it is well known that female scholars tend to be more involved in “academic housework” than their male peers, spending time on “feminine tasks” such as student counseling and organizational duties^51–53^. Therefore, the gendered organization of academic work and the unbalanced division of caregiving tasks generate an accumulation of disadvantages for female scholars.

Although none of the interviewees have given up their jobs in academia, most of the female respondents reported professional costs as a result of caregiving for the older people and adults with disabilities (Fig. 2). For example, women scholars pointed out that as they are the ultimate caregivers for their older people, they found it difficult to do fieldwork or go on research stays abroad, which ultimately compromised their research, networking and academic collaborations. Angervall and Hammarfelt^54^ called to rethink the requirements of career mobility in higher education since it tends to perpetuate gender inequality. Our research demonstrates that when caregiving duties mainly rely on women in society, the requirement of career mobility adds an unnecessary burden on female scholars.

Interestingly, the narratives of female interviewees resonated with the “superhero” lexicon^29,48^ since they reported not compensating for the caregiving time at work but rather carrying out all caregiving and work-related duties. Instead of reducing the time spent at work, interviewees gave up leisure, rest, and social relationships, which ultimately hindered their well-being.

Moreover, interviewees suggested that the flexible working hours characteristic of academia can be an advantage since this flexibility allows them to carry out older people’s care while conducting their full-time jobs. Yet, the same interviewees pointed out that this flexibility is a “double-edged sword” because caregiving duties fall on them even though there are other family members who could be responsible for the older people, something known in the literature as the “flexibility paradox”^55^.

### No possible solutions when something remains invisible

When a phenomenon remains invisible, it does not require solutions. Although the “double presence” phenomenon in academia that highlights the double role of women as mothers and scholars has been extensively researched [e.g.^3,4,48^], the “triple presence” that adds a new role as caregivers of the older people and adults with disabilities remains unstudied, unnamed, and invisible. As an invisible phenomenon, it is not even considered as a source of gender inequity in the institutional sphere, in the research centers and universities. The academic institutions offer no solutions to help caregivers of the older people and adults with disabilities in Spain, only the extension of contracts under certain circumstances. The Law 14/2011 of 1 June 2011 on Science, Technology, and Innovation in Spain (revised text, last amended 11 January 2023)^56^ allows for the extension of contracts in various circumstances, including care for dependent persons. However, it is not clear under which circumstances, and under which type of contract this rule applies. To close the gender gap in science, new policies are needed to build a more egalitarian and heterogeneous scientific community and society^18^. Raising awareness on the issue of caring for older people is one of the first actions and academia has the responsibility to do so as a role model for transformation towards an egalitarian, just and equitable future. This necessarily requires radical changes in the structures of academia itself^57^. With this research, we have shed light on a few solutions to foster gender equity in academia in the context of caregiving for the older people, such as mental support, work leave paid periods and institutionalizing care centers for the older people in academic institutions.

We hope that with this research we spark a dialogue that considers the extra layer of gender inequity in academia, that is, caregiving for older people and adults with disabilities, a phenomenon that, despite being unnamed, is inescapable in our society.

### Research limitations

First, the research was initiated in the context of the Gender and Science group of the Iberian Association of Limnology, and, therefore, the results might have a particular bias towards women’s perspectives and scholars familiarized with discussions around gender equity. Through the snowball technique, we included the perspectives and experiences of male scholars and other scientists who are not familiar with the narratives of gender equity in academia. However, future studies should assess how other aspects such as class, national or cultural origin or career status intersect with gender on the impacts of caregiving for researchers.

Second, given that interviewees and authors of this study belong to the Spanish scientific communities of environmental sciences and sustainability, some of the conclusions of this research might not be extrapolated to other scientific systems. Previous research has shown that geographic contexts, cultural background, race/ethnicity, or access to socio-economic resources (e.g. education and income) influence the caregiving experiences, support services, and caregiver health [e.g.^58–60^]. However, the study is relevant in the context of environmental sciences and can be extrapolated to many experimental scientific disciplines, given that it involves significant research in the field that requires the temporary absence of the caregiver from the family environment. Despite our research being centered on a specific profile of people (i.e. Spanish scientists in the fields of environmental science and sustainability), our ultimate intention was not to comprehensively understand the geography of caregiving to older adults and people with disabilities, but rather to draw the attention of scholars to investigate whether the “triple presence” phenomenon, which uncovers a new role of scientists as caregivers of older adults and people with disabilities, is present in other academic systems.

## Supplementary Information

Below is the link to the electronic supplementary material.

Supplementary Material 1

## Data Availability

The datasets generated and analysed during the current study are not publicly available due to the confidentiality required in the interviews conducted but are available from the corresponding author on reasonable request. Data generated during this study that does not affect confidentiality clauses are included in their supplementary information files.
