# Supplementary material for: Older people care increases the gender gap in academia
Source: Sci Rep. 2025 Sep 29;15:33336. doi: 10.1038/s41598-025-13360-1 (PMC12480459; doi:10.1038/s41598-025-13360-1)
Supplement: Supplementary file 1 — Supplementary Material 1 [file 41598_2025_13360_MOESM1_ESM.pdf]

Supplementary Information

# OLDER PEOPLE CARE INCREASES THE GENDER GAP IN ACADEMIA

**María Rosario Vidal-Abarca[1*], Berta Martín-López[2], Anna Sala-Bubaré[3], María Anton-Pardo[4], Nuria Catalan[5], Anna Freixa[6], Anna Lupon[5], Nestor Nicolás-Ruiz[1], Silvia Poblador[7], Pablo Rodríguez-Lozano[8], María del Mar Sánchez-Montoya[9], María Luisa Suárez[1].**

[*]Corresponding Author. e-mail: charyvag@um.es

[1]Department of Ecology and Hydrology University of Murcia. Murcia (Spain).

Supplementary Information

**Table S1**. Personal and professional profile of the interviewees. (? = indicates missing information). AGM= aunt-grandmother; B= brother; F= father; FL = father-in-law; FP= female partner; GF= grandfather; GM= grandmother; M= mother; ML= mother-in-law; S= sister; U= uncle

| Code | Position | Institution | Age | Civil status | Gender | Children | | who do they care for? | Leisure | Interview time (minutes) |
|---|---|---|---|---|---|---|---|---|---|---|
| | | | | | | Number | Age | | Time (h/week) | |
| W01 | Professor (Full) | University | 42 | Single | Woman | 0 | 0 | M | 17 - 18 | 29:00 |
| M02 | Professor | University | 74 | Married | Man | 2 | 44, 46 | FP | 2 | 43:52 |
| W03 | Professor | University | 65 | Single | Woman | 0 | 0 | M, U, S | 1 | 29:52 |
| M04 | Professor | University | 57 | Divorced | Man | 2 | 23, 30 | M | 10 | 32:23 |
| W05 | Professor | University | 40 | Single | Woman | 0 | 0 | M | 5 | 25:42 |
| W06 | Professor | University | 60 | Divorced | Woman | 2 | 28, 30 | M | 3 | 21:12 |
| W07 | Professor | University / Research institute | 60 | Married | Woman | 1 | 23 | M, F | 7 | 33:12 |
| M08 | Pre-doctoral researcher | University | 30 | Single | Man | 0 | 0 | F | 3:30 | 29:45 |
| M09 | Professor | University | 45 | Single | Man | 0 | 0 | F | 5 | 17:21 |
| M10 | Professor | University | 60 | Domestic partner | Man | 2 | 17, 18 | F, M | 3 | 13:28 |
| M11 | Postdoctoral researcher | University | 35 | Single | Man | 0 | 0 | F, M | 0 | 29:09 |
| W12 | Professor | University | 42 | Domestic partner | Woman | 2 | 11, 15 | F, M | 1 | 15:12 |
| W13 | Professor | University | 59 | Married | Woman | 0 | 0 | M | 4 | 16:58 |
| M14 | Professor | University | 49 | Domestic partner | Man | 3 | 2, 15, 15 | F, M | 0 | 26:24 |
| M15 | PhD student | University | 28 | Single | Man | 0 | 0 | GF, GM | 4 | 25:43 |
| W16 | Full scientist | Research institute | 67 | Single | Woman | 0 | 0 | M | 3 - 4 | 18:21 |
| W17 | Teacher | University | 61 | Married | Woman | 1 | 28 | F, M, B | 0 | 18:27 |
| W18 | Postdoctoral researcher | University | 43 | Single | Woman | 0 | 0 | AGM | 3 | 18:27 |
| W19 | Researcher | Research institute | 60 | Married | Woman | 2 | 14, 19 | F, M | 4 | 28:29 |
| W20 | Researcher | University | 38 | Domestic partner | Woman | 0 | 0 | F,M,FL,ML | 3 | 33:29 |
| W21 | Professor | University | 58 | Domestic partner | Woman | 2 | 21, 24 | F, M | 5 | 23:07 |
| W22 | Cultural manager | Town Council | 30 | Single | Woman | 0 | 0 | M | 20 | 35:26 |

| | | | | | | | | | | |
|---|---|---|---|---|---|---|---|---|---|---|
| W23 | Postdoctoral researcher | University | 39 | Single | Woman | 0 | 0 | M | 3 | 16:21 |
| W24 | Professor | University | 40 | Single | Woman | 0 | 0 | M | 0 | 23:51 |
| W25 | Professor | University | 59 | Married | Woman | 1 | 27 | M | 3:30 | 10:29 |
| M26 | Professor | University | 57 | Domestic partner | Man | 2 | 7, 10 | M | 3 | 17:04 |
| M27 | Professor | University | 63 | Domestic partner | Man | 0 | 0 | M | 5 | 12:46 |
| M28 | Postdoctoral researcher | University | 45 | Married | Man | 3 | 8, 11, 13 | GF, GM | 0 | 11:17 |
| W29 | Professor | University | 67 | Married | Woman | 3 | 25, 35, 40 | F, M | 3 | 19:53 |
| W30 | Professor | University | 58 | Single | Woman | 1 | 22 | M | 0 | 20:11 |
| W31 | Professor | University | 64 | Married | Woman | 3 | >25 | M | 3:30 | 23:24 |
| M32 | Professor | University | 59 | Married | Man | 2 | 14, 16 | M | 6 | 23:36 |
| W33 | Professor | University | 60 | Single | Woman | 0 | 0 | F, M | 0 | 30:13 |
| W34 | Professor | University | 65 | Single | Woman | 0 | 0 | M | 7 | 21:24 |
| W35 | Professor (Associate) | University | 47 | Domestic partner | Woman | 1 | 13 | F | 2 | 25:60 |
| W36 | Postdoctoral researcher | Research institute | 37 | Domestic partner | Woman | 0 | 0 | F | ? | 44:14 |

**Interview protocol**

**Text S1.** Interview guide in English

(1) Personal data and about his life story

Age

Gender

Civil status

Place of residence

University or research center

Number of children and their ages

Leisure activities

(2) Description about the older person or people you care for

Who do you care for?

How much time do you dedicate to their care?

Do you feel prepared for care?

Are you satisfied with the care you provide?

(3) About the help received both in the family environment and in the workplace for the care of the older adult

Do you have help from family members to care for the older adult?

Whose?

What role do they play in your co-workers during care?

Does your Institution facilitate your dedication to caring for the older adult?

(4) On the physical, psychological and professional costs of caring for the older adult

How has caring for the older adult affected your physical and/or emotional health?

And your activity at work?

Has it affected your personal promotion?

(5) About other personal costs of older care

Has caring for the older adult entailed financial costs for you?

And social costs?

And other costs?

(6) Any more comments?

**Text S2**. Interview guide in Spanish

(1) Datos personales y sobre su historia de vida

Edad

Género

Estado civil

Lugar de residencia

Universidad o Centro de Investigación

Número de hijos y sus edades

Actividades de ocio

(2) Descripción de la persona o personas mayores a las que está cuidando o ha cuidado

¿Quién es la persona cuidadora?

¿Cuánto tiempo dedica a su cuidado?

¿Te sientes preparado/a para llevar a cabo la tarea del cuidado?

¿Estás satisfecho/a con tu tarea de cuidador/ra?

(3) Sobre la ayuda recibida tanto en el entorno familiar como en el laboral durante el cuidado de las personas mayores

¿De quién?

¿Qué papel han desempeñado sus colegas durante los cuidados?

¿Facilita su Institución su dedicación al cuidado de las personas mayores?

(4) Sobre los costes físicos, psicológicos y profesionales del cuidado de personas mayores

¿Cómo ha afectado el cuidado a su salud física y/o emocional?

¿Y a su actividad laboral?

¿Ha afectado a su promoción personal?

(5) Sobre los costes personales del cuidado de mayores

¿Te ha supuesto costes económicos el cuidado de la persona mayor?

¿Y costes sociales?

¿Y otros costes?

(6) ¿Desea hacer algún comentario más?
